# Detection of Measles Virus Genotype B3, India

**DOI:** 10.3201/eid2010.130742

**Published:** 2014-10

**Authors:** Vijesh S. Kuttiatt, Sanughosh Kalpathodi, Sindhu T. Gangadharan, Lalitha Kailas, Easwaran Sreekumar, Suja M. Sukumaran, Radhakrishnan R. Nair

**Affiliations:** Rajiv Gandhi Centre for Biotechnology, Thiruvananthapuram, Kerala, India (V.S. Kuttiatt, S. Kalpathodi, E. Sreekumar, S.M. Sukumaran, R.R. Nair);; Sree Avittom Thirunal Hospital, Government Medical College,Thiruvananthapuram, Kerala (S.T. Gangadharan, L. Kailas)

**Keywords:** measles, virus, genotype B3, Kerala, India

**To the Editor:** Molecular epidemiologic investigations and virologic surveillance contribute notably to the control and prevention of measles (*1*). Nearly half of measles-related deaths worldwide occur in India, yet virologic surveillance data are incomplete for many regions of the country (*2*,*3*). Previous studies have documented the presence of measles virus genotypes D4, D7, and D8 in India, and genotypes D5, D9, D11, H1, and G3 have been detected in neighboring countries (*3*,*4*).

Kerala, India’s southernmost state, has high measles vaccination coverage compared with many other states in the country; however, the disease is still endemic in the region. Two districts, Thiruvananthapuram and Malappuram, report the highest numbers of cases (*5*). Baseline data on circulating measles virus genotypes are needed for measles elimination, but such data are not available for Kerala. In this context, we performed a pilot genetic analysis of the measles virus strains circulating in Thiruvananthapuram, the capital of Kerala. We used throat and nasopharyngeal swab and serum samples from children admitted to Sree Avittom Thirunal Hospital during measles outbreaks occurring March–August 2012. 

We used the Vero/human-SLAM cell line (http://www.phe-culturecollections.org.uk) for isolation of measles virus from throat and nasopharyngeal swab samples. For serologic confirmation of cases, we used a commercial measles IgM ELISA kit (IBL International GmbH, Hamburg, Germany). Virus genotyping was based on the 450-nt coding sequence for the carboxyl terminus of nucleoprotein (N) of measles virus, as recommended by the World Health Organization (*3*,*6*). We extracted RNA from the samples using TRIzol reagent (GIBCO-BRL, Grand Island, NY, USA). We performed reverse transcription PCR using a SuperScript One-Step RT-PCR kit with a Platinum *Taq* system (Invitrogen, Carlsbad, CA, USA) and previously described primers (*3*,*6*). Amplicons were subjected to bidirectional sequencing using a BigDye Terminator v3.1 cycle sequencing kit (Applied Biosystems, Foster City, CA, USA). We edited and aligned nucleotide sequences using Bio Edit 7.1.11 software (*7*). Phylogenetic analysis was performed by using the maximum-likelihood method implemented in the MEGA5 program (*8*) to compare the determined N gene sequences with the World Health Organization reference sequences of the 24 known measles genotypes.

PCR products could be amplified from 16 of the 24 samples analyzed. Ten samples provided high quality sequence reads for the N gene coding region, which were used for further analysis. Clinical and demographic data for these 10 cases, virus isolation status, and GenBank accession numbers of the sequences are summarized in the Table. 

**Table:** Details of the clinical samples used for genotype analysis of measles virus strains, Kerala, India, 2012

Strain name*	Clinical details of patients					GenBank accession no.
	Age	Sex	Measles vaccination status	Contact history	Complications	
MVi/Thiruvananthapuram.IND/12.12[B3]	11 mo	F	No	Yes	Pneumonia	KC997602
MVs/Thiruvananthapuram.IND/14.12/1[B3]	9 mo	M	No	Yes	Pneumonia	KC997603
MVs/Thiruvananthapuram.IND/14.12/2[B3]	3 y	M	Yes	Yes	None	KC997604
MVi/Thiruvananthapuram.IND/14.12/3[B3]	6 y	M	No	No	None	KC997605
MVs/Thiruvananthapuram.IND/14.12/4[B3]	7 mo	M	No	Yes	Pneumonia	KC997606
MVi/Thiruvananthapuram.IND/15.12[B3]	10 mo	M	Yes	Yes	None	KC997607
MVs/Thiruvananthapuram.IND/30.12[B3]	7 mo	F	No	No	Pneumonia	KC997608
MVs/Thiruvananthapuram.IND/31.12/1[B3]	9 mo	F	No	Yes	Pneumonia	KC997609
MVs/Thiruvananthapuram.IND/31.12/2[B3]	2 y	F	No	Yes	Pneumonia	KC997610
MVs/Thiruvananthapuram.IND/34.12[D8]	10 y	M	No	No	None	KC997611

*Named according to World Health Organization nomenclature following the scheme: measles virus (MV), isolate (i) or clinical specimen (s)/place. country/epidemiologic week.year/sample identity [genotype].

Phylogenetic analysis revealed 1 of the 10 measles virus strains to be of genotype D8 (Technical Appendix Figure 1), a genotype previously found to be circulating in Kerala and in other regions of India (*3*,*6*,*9*,*10*). The other 9 virus strains were closely related to B3 genotype reference strains, indicating circulation of the B3 genotype in Kerala (Technical Appendix Figure 1). The nucleotide sequences of 7 of the 9 strains were identical, indicating a single chain of transmission. The remaining 2 samples showed sequence divergence, indicating independent sources of infection. In a phylogenetic analysis comparing the Kerala B3 genotypes and a dataset of global measles B3 genotypes selected from GenBank, the strains from Kerala formed a separate cluster (Technical Appendix Figure). This cluster also contains a strain from Germany (MVs/Regensburg.DEU/37.12/). The strains in this cluster show close identity to a measles strain identified in the state of New York, USA. A search of the MeaNS (Measles Nucleotide Surveillance) database revealed that the Kerala B3 sequences had the closest match to the strain isolated in Germany mentioned above and also to a strain from the Sultanate of Oman (MVs/Muscat.OMN/38.11/).

The B3 genotype identified in this study could be a previously undetected genotype endemic to India or a recent importation. B3 is endemic to many countries in Africa, and its importation into Europe and North America and elsewhere has been described (*4*). Further studies with samples from a wider geographic area of Kerala are required to determine the spread and genetic diversity of these strains and ascertain their relationship to the global B3 strains. It would also be of interest to determine whether B3 strains co-circulate with D8 strains, whether they will eventually replace D8 as the predominant genotype in Kerala, or if they will cease to exist as the outbreak diminishes. This report underscores the need for systematic nationwide measles virus surveillance in India to identify all endemic virus genotypes and to monitor importation of new virus strains from other countries.

This study was approved by the Institutional Human Ethics Committee of the Rajiv Gandhi Centre for Biotechnology. It was carried out as part of the National Virology Network Laboratory Program of the Indian Council of Medical Research, which provided financial support. 

Technical Appendix We used measles virus genotype B3 sequences detected globally to analyze origins of strains identified in India.
