# Supplementary material for: Detection of Measles Virus Genotype B3, India
Source: Emerg Infect Dis. 2014 Oct;20(10):1764–6. doi: 10.3201/eid2010.130742 (PMC4193266; doi:10.3201/eid2010.130742)
Supplement: Technical Appendix — We used measles virus genotype B3 sequences detected globally to analyze origins of strains identified in India. [file 13-0742-Techapp-s1.pdf]

DOI: http://dx.doi.org/10.3201/eid2010.130742

Detection of Measles Virus Genotype B3, India

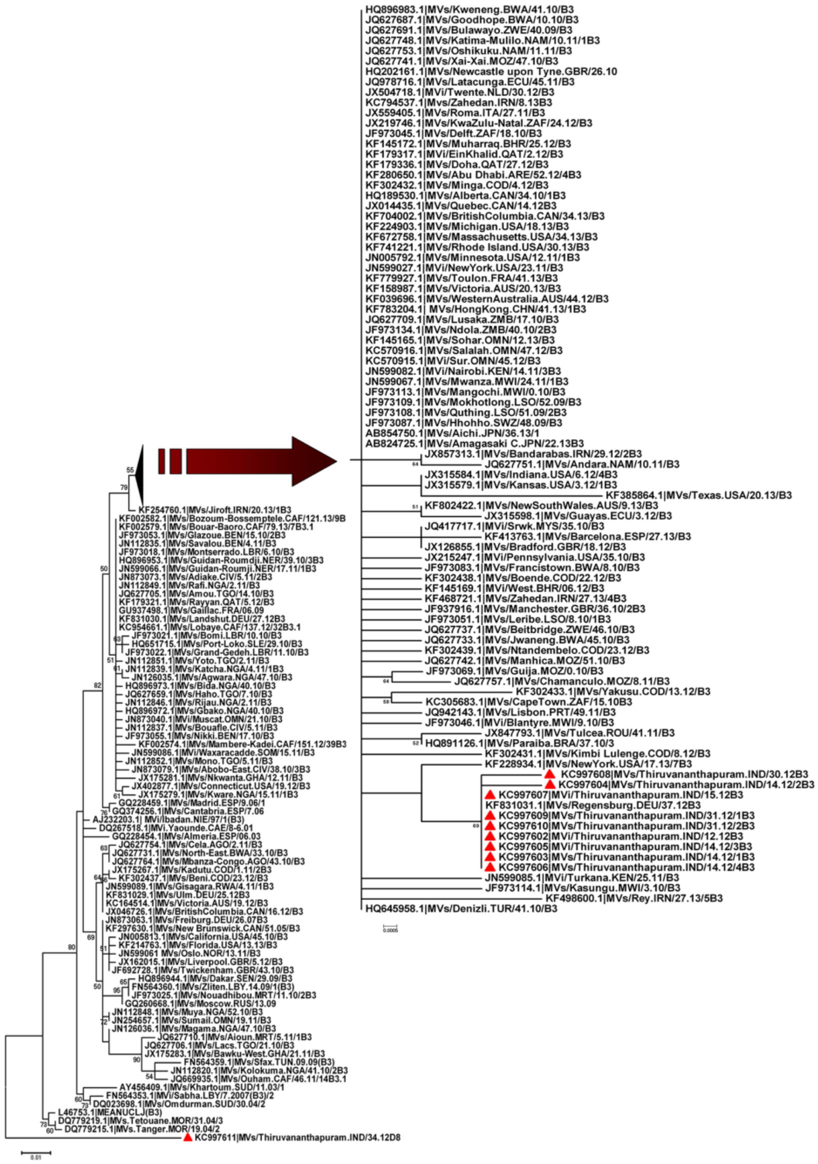

Technical Appendix Figure. Phylogenetic analysis of B3 genotype measles virus sequences detected in India with selected global B3 genotypes. A D8 genotype sequence from Thiruvananthapuram was used as the outgroup. We performed maximum-likelihood analysis with 1,000 bootstrap replications using Kimura2-parameter correction with gamma-distributed settings. Solid red triangles indicate strains from the current study; scale bar indicates nucleotide substitutions per site.
